# Bilateral Distal Radius Fractures in a 12-Year-Old Boy after Household Electrical Shock: Case Report and Literature Summary

**DOI:** 10.1155/2014/235756

**Published:** 2014-01-05

**Authors:** Norman Stone, Mara Karamitopoulos, David Edelstein, Jenifer Hashem, James Tucci

**Affiliations:** Department of Orthopaedic Surgery, Maimonides Medical Center, 927 49th Street, 2nd floor, Brooklyn, NY 11219, USA

## Abstract

*Background*. Fracture resulting from household electric shock is uncommon. When it occurs, it is usually the result of a fall; however, electricity itself can cause sufficient tetany to produce a fracture. We present the case of bilateral fractures of the distal radii of a 12-year-old boy which were sustained after accidental shock. The literature regarding fractures after domestic electric shock is also reviewed. *Methods*. An Ovid-Medline search was conducted. The resultant articles and their bibliographies were surveyed for cases describing fractures resulting from a typical household-level voltage (110–220 V, 50–60 Hertz) and not a fall after the shock. Twenty-one articles describing 22 patients were identified. *Results*. Twenty-two cases were identified. Thirteen were unilateral injuries; 9 were bilateral. Proximal humerus fractures were most frequent (8 cases), followed by scapula fractures (7 cases), forearm fractures (4 cases), femoral neck fractures (2 cases), and vertebral body fracture (1 case). Eight of the 22 cases were diagnosed days to weeks after the injury. *Conclusions*. Fracture after electric shock is uncommon. It should be suspected in patients with persistent pain, particularly in the shoulder or forearm area. Distal radius fractures that occur during electrocution are likely due to tetany.

## 1. Introduction

Electricity can damage human tissue in the 4 following ways [1–9]:

1. disruption of physiologic conduction systems, including cardiac contraction and diaphragm excursion, leading to arrhythmia and apnea;
2. thermal energy generated by the electrical current;
3. electroporation of cell membranes occurs leading to a disruption of intracellular ion and protein balance, and ultimately, apoptosis;
4. mechanical injury due to a fall or forceful muscle contraction.

The degree of electrical injury is dependent on the currant, voltage, duration of contact, tissue resistance, and the path of the current flow through the body [1, 3, 4].

Initial medical care after electrical injury focuses on the most common sequellae of electrocution: infection of the burn wounds, myonecrosis leading to acute renal failure, cardiac arrest or arrhythmia, pneumonia, nausea, and vomiting [2, 5]. Whereas fracture during electroconvulsive therapy is a well-established complication described in the psychiatry literature [10–12], fracture after accidental electrical injury is uncommon. When it does occur, it is usually the result of a fall sustained after the shock. Additionally, fractures can occur as a consequence of uncontrolled muscular contraction. A well-described example of this kind of injury during electrocution occurs with posterior shoulder dislocations, in which the humeral head is forced posteriorly and superiorly against the acromion, and medially against the glenoid fossa, due to the powerful shoulder girdle musculature. As a result, the humeral head dislocates and then becomes impacted against the bony posterior glenoid rim. This motion results in a fracture defect of the anterior humeral head just medial to the lesser tuberosity (reverse Hill-Sachs) [13, 14].

However, it is possible that the electricity itself can occasionally cause sufficient tetany to produce a fracture. We present the case of bilateral, apex-dorsal, buckle fractures of the distal radii of a 12-year-old boy which were sustained after accidental shock by a faultily-wired apartment entrance door. We further review the literature regarding fractures after domestic electric shock where the fracture appears to have occurred because of the electricity itself.

## 2. Case Report

**Figure 1:**
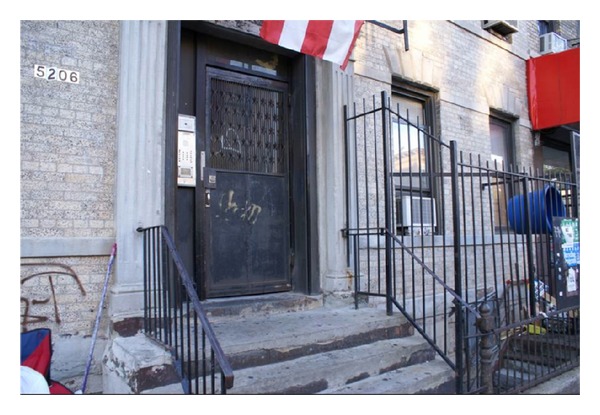
The offending door and stair railing.

A 12-year-old boy presented to the Pediatric Emergency Department shortly after receiving an electric shock at a nearby apartment building. As the boy, his mother, and younger brother were waiting outside the building for friends to “buzz them in” (Figure 1), an individual exited the building and held the door open. The mother and younger brother entered without touching the door. As our patient entered the building, he grasped the metal door knob with his left hand while at the same time keeping his right hand on a metal stair railing that was adjacent to the entrance. This action occurred just as the door entry mechanism was activated from upstairs. As a result, the patient sustained an electrical shock inducing upper extremity tetany, prohibiting him from releasing his grip. The shock lasted approximately 5 seconds and was witnessed by the patient's mother. The patient retained consciousness throughout the event and did not fall afterwards.

Initial evaluation in the Emergency Department centered on the potential cardiac, myopathic, and renal aspects of the injury. The patient was admitted for telemetry monitoring in the Pediatric Intensive Care Unit. No arrhythmias were noted. The following morning, the patient complained of bilateral wrist pain. He had mild swelling and erythema, therefore an orthopaedic surgery consult was requested for evaluation.

**Figure 2:**
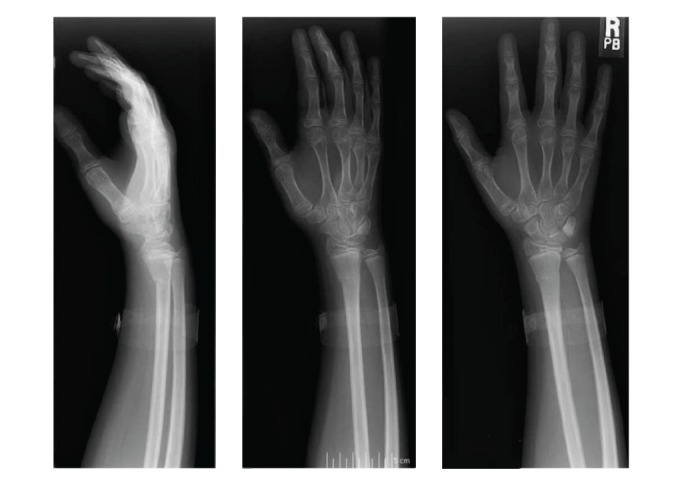
Radiographs of right wrist showing apex-dorsal distal radius buckle fracture.

**Figure 3:**
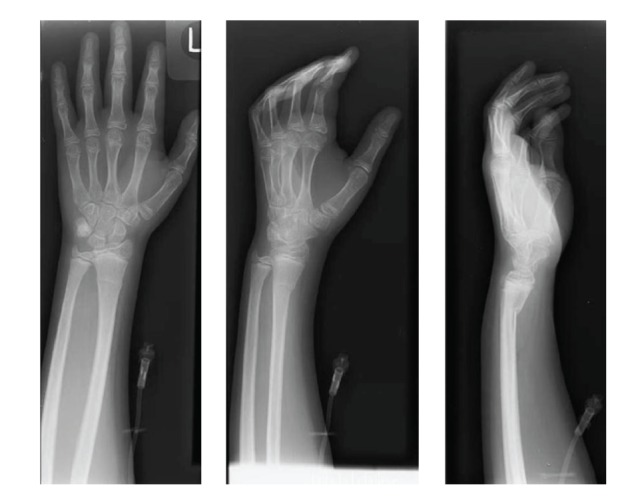
Radiographs of left wrist showing apex-dorsal distal radius buckle fracture.

The child was able to use both hands, favoring the right over the more painful left. There were no burn marks or abrasions, but mild, bilateral, distal radius volar angulation deformities were present. Anteroposterior, lateral, and oblique radiographs of both wrists revealed bilateral, buckle-type, apex-dorsal angulated fractures of the distal radial metaphyses (Figures 2 and 3). The patient was placed in bilateral volar splints to reduce wrist motion, preserve hand function, and permit examination of his skin. He was discharged home directly from the PICU on hospital day three and followed an uneventful course to clinical and radiographic healing.

## 3. Discussion

### 3.1. Most Common Fractures after Electrical Injury

Our literature review identified several case reports describing fractures after electrical injury, but no attempts to inventory all published fractures resulting from domestic electrocution. We identified 21 articles describing 22 cases where a fracture was the result of the electricity itself and not a fall at the time of injury. Nineteen articles were published in English language journals. Two were published in a different language but had English language abstracts available.

Overall, fractures were reported most frequently in the proximal humerus and scapula, as part of the posterior shoulder fracture-dislocation injury pattern. Next most common were forearm fractures, followed by femoral neck and vertebral body fractures (Table 1).

### 3.2. Forearm Fractures after Electrical Injury

We identified four cases in addition to our patient [16, 22–24] of isolated forearm fractures after household electrical injury. Interestingly, three of the four involved 11-year-old girls [16, 22, 23]. Three cases were unilateral [22–24], and one described bilateral injuries [16]. Hostetler and Davis [23] described a girl who grasped the metal doorknob of a lifeguard shack while standing in pooled water. The power cord of a nearby radio was routed under the same door and ran through the same puddle; the girl sustained a unilateral Galeazzi fracture during a 5–10 second tetanic grasp. Similarly, Adams and Beckett [16] recorded the story of a girl who sustained bilateral distal radius buckle fractures while simultaneously switching on the overhead light and grasping a metal hand railing of an outdoor shed. Tucciarone et al. [22] reported a girl who incurred an “incomplete” distal radius fracture who presented with throbbing and tingling in both arms. Finally, Evans and Little [24] described the case of an 84-year-old woman who touched a puddle of water which was in contact with a faulty wire that was involved in an electrical fire; she subsequently sustained a distal radius fracture.

Authors have proposed that the posterior shoulder fracture-dislocation pattern of injury is produced by forceful, sustained, posterior-directed tetany of the deltoid, latismus dorsi, teres major, teres minor, and infraspinatus muscles [25–27]. It is believed that the severity of these injuries occur as a continuum that results from the intensity and duration of muscular contraction. Once the humeral head dislocates posteriorly, the anterior fracture-defect results from the subsequent impaction of the humeral head against the glenoid rim. If the tetany ceases, this remains the extent of injury. However, with further insult the fracture will continue to propagate as the tendinous attachments produce shearing forces to the bone. The humeral head is subsequently avulsed off along with the greater and lesser tuberosities. With continued contraction, the triceps, coracobrachialis, biceps, and deltoid muscles force the humeral shaft fragment superiorly against the acromion, causing further comminution. In this circumstance, it is this combination of both indirect and direct means by which the powerful muscular contractions cause such extensive damage [14].

Our patient, as well as the four other reported patients with forearm fractures, sustained apex-dorsal distal radius fractures. Extrapolating from the shoulder fracture-dislocation injury pattern, we suggest that tetanic contraction of flexor carpi radialis and flexor carpi ulnaris directly may produce a moment of sufficient magnitude at the distal radius to produce the observed apex-dorsal fracture pattern, similar to shearing forces experienced during fracture-dislocations.

### 3.3. Delay in Diagnosis

Several authors describe delays in diagnosis of a fracture after electrical injury of days [15–18] or weeks [19–21] after injury. This likely seems attributable to a delay in presentation of the patient, investigation of potentially greater comorbid sequelae, and the challenge in obtaining a clear history and physical examination on a recently electrocuted patient. Our patient was not diagnosed until his second hospital day. His physical examination demonstrated minimal deformity and he did not report significant pain at his wrists. Thus, his forearms were not imaged until orthopaedic consultation.

## 4. Summary

After electrical injury, care is appropriately focused on the potentially high morbidity sequelae of the injury: cardiac insult leading to arrhythmia, cutaneous burns, and myonecrosis leading to renal failure. Fracture after electrical injury is uncommon and is often not diagnosed until days or weeks after injury. When it does occur, it most frequently involves the shoulder. The mechanism is likely due to the sustained tetanic contraction of powerful muscle groups—the superoposterior muscles of the shoulder and wrist flexors at the forearm. Because the mechanism by which fractures occur can be quite complex, it is possible that more fractures than previously believed are attributable to tetanic contractures and not the result of a fall itself. Unfortunately, in these circumstances it remains difficult if not impossible to differentiate between cause and effect. Therefore, heightened sensitivity to deformity and patient complaints about persistent pain after injury should be evaluated with appropriate radiographs in order to rule out fracture.

## Figures and Tables

**Table 1:** Anatomic distribution of reported fractures after household-voltage electrical injury.

Bone	Bilateral	Unilateral	Total fractures	References
Forearm				
Colles		1	1	[22]
Galeazzi		1	1	[23]
Greenstick	1		2	[16]
Distal radius		1	1	[24]
Shoulder				
Proximal Humerus	3	5	11	[15, 20, 26, 28–31]
Scapula	4	3	11	[18, 19, 25, 32–35]
Vertebrae				
Lumbar L4 burst		1	1	[17]
Femur				
Neck	1	1	3	[36, 37]
